# Use of a Smartphone-Based Medication Adherence Platform to Improve Outcomes in Uncontrolled Type 2 Diabetes Among Veterans: Prospective Case-Crossover Study

**DOI:** 10.2196/44297

**Published:** 2023-08-10

**Authors:** Amneet Rai, Mark Riddle, Rajendra Mishra, Nhien Nguyen, Kelly Valine, Megan Fenney

**Affiliations:** 1 Veterans Affairs Sierra Nevada Healthcare System Reno, NV United States; 2 University of Nevada Reno School of Medicine Reno, NV United States

**Keywords:** application, biguanide, blood glucose, case-crossover, diabetes management, diabetes, diabetic, digital health intervention, epidemiology, glucose monitoring, glucose, HbgA1c, hemoglobin A1c, hemoglobin, hyperlipidemia, hypertension, interquartile range, IQR, MAS, Medication Adherence Scores, medication adherence, mobile health intervention, mobile phone, odds ratio, paired t test, quasi experimental, SEAMS, Self-Efficacy for Appropriate Medication Use Scale, smartphone, sulfonylurea, T2DM, Type 2 Diabetes Mellitus, veteran

## Abstract

**Background:**

Medication nonadherence is a problem that impacts both the patient and the health system.

**Objective:**

The objective of this study was to evaluate the impact of a novel smartphone app with patient-response-directed clinical intervention on medication adherence and blood glucose control in noninsulin-dependent patients with type 2 diabetes mellitus (T2DM).

**Methods:**

We enrolled 50 participants with T2DM not on insulin with smartphones from a rural health care center in Northern Nevada for participation in this case-crossover study. Participants underwent a standard of care arm and an intervention arm. Each study arm was 3 months long, for a total of 6 months of follow-up. Participants had a hemoglobin A_1c_ (HbA_1c_) lab draw at enrollment, 3 months, and 6 months. Participants had monthly “medication adherence scores” (MAS) and “Self-Efficacy for Appropriate Medication Use Scale” (SEAMS) questionnaires completed at baseline and monthly for the duration of the study. Our primary outcomes of interest were the changes in HbA_1c_ between study arms. Secondary outcomes included the evaluation of the difference in the proportion of participants achieving a clinically meaningful reduction in HbA_1c_ and the difference in the number of participants requiring diabetes therapy escalation between study arms. Exploratory outcomes included the analysis of the variation in medication possession ratio (MPR), MAS, and SEAMS during each study arm.

**Results:**

A total of 30 participants completed both study arms and were included in the analysis. Dropouts were higher in participants enrolled in the standard of care arm first (9/25, 36% vs 4/25, 16%). Participants had a median HbA_1c_ of 9.1%, had been living with T2DM for 6 years, had a median age of 66 years, and had a median of 8.5 medications. HbA_1c_ reduction was 0.69% in the intervention arm versus 0.35% in the standard of care arm (*P*=.30). A total of 70% (21/30) of participants achieved a clinically meaningful reduction in HbA_1c_ of 0.5% in the app intervention arm versus 40% (12/30) in the standard of care arm (odds ratio 2.29, 95% CI 0.94-5.6; *P*=.09). Participants had higher odds of a therapy escalation while in the standard of care arm (18/30, 60% vs 5/30, 16.7%, odds ratio 4.3, 95% CI 1.2-15.2; *P*=.02). The median MPR prior to enrollment was 109%, 112% during the study’s intervention arm, and 102% during the standard of care arm. The median real-time MAS was 93.2%. The change in MAS (1 vs –0.1; *P*=.02) and SEAMS (1.9 vs –0.2; *P*<.001) from baseline to month 3 was higher in the intervention arm compared to standard of care.

**Conclusions:**

A novel smartphone app with patient-response-directed provider intervention holds promise in the ability to improve blood glucose control in complex non–insulin-dependent T2DM and is worthy of additional study.

## Introduction

Medication nonadherence is a problem that impacts both the patient and the health system. Medication nonadherence results in undesirable clinical outcomes, especially in patients with chronic diseases, as well as increases in health care resource consumption [1]. The International Society of Pharmacoeconomics and Outcomes Research (ISPOR) has identified that 33%-69% of medication-related hospitalizations are due to poor adherence, accounting for up to US $100 billion in annual health care costs [2,3].

Specifically, medication nonadherence has been identified as a major problem in the management of patients with type 2 diabetes mellitus (T2DM). Studies have shown only 40%-60% of patients maintain a proportion of days covered (PDC), defined as the proportion of days over a given period that a patient has medication available to take, of ≥80%, and only 45% of patients with T2DM are able to maintain glycemic control during treatment [1,4,5]. In patients with T2DM, nonadherence has been correlated with poor glycemic control, exposing patients to significant long-term complications of the disease [4]. Improvements in medication adherence in this population have been shown to improve clinical outcomes as well as reduce health care costs [6,7].

Specific to the “veteran” population, T2DM affects greater than 20% of veterans compared to 8% of the general population [8]. Studies have shown that 73% of veterans are nonadherent with their medications [8]. The number of medications and complexity of their medication regimen were associated with increased rates of nonadherence [8].

Thus, with the known importance of medication adherence in problems such as T2DM, considerable research efforts have been put forth to improve adherence and attendant health outcomes. Advances in technology have been shown to improve medication adherence in a wide range of disease states [9-20]. Automated alerts have been shown to improve medication adherence in patients with hypertension [10]. Smartphone apps have demonstrated increased patient awareness and improved medication adherence in T2DM [9,15,17]. Automated reminders, along with pharmacist intervention, have improved glycemic control as well as medication adherence in patients with diabetes [6,11]. A limitation of all these studies is the use of “medication possession ratio” (MPR), PDC, or “medication administration score” (MAS) as a surrogate marker for medication adherence. These measures have significant limitations and bias toward an overestimation of true adherence. Current measures also lack auditable information that could be reviewed by the patient care team in directing interventions. Due to such limitations with current technologies, many of these studies have failed to demonstrate clinically meaningful improvement in patients’ control of chronic disease states [6,9,10]. Within the veteran population, continued challenges remain in improving medication adherence and the attendant morbidity of uncontrolled diabetes.

The purpose of this study was to operationalize a pragmatic study design to evaluate the impact of a novel smartphone app that provides customizable medication alerts, real-time auditable adherence data, and a novel engagement system on medication adherence and blood glucose control in a single-site health care system.

## Methods

### Study Design

In a pragmatic, quasi-experimental pilot study approach, we employed a case-crossover design where participants underwent both a standard of care arm as well as an intervention arm. Study participants were assigned in alternating blocks of 5 to either a standard of care arm followed by intervention or intervention followed by standard of care. Each study arm was 3 months long, for a total of 6 months of follow-up. Participants had a hemoglobin A_1c_ (HbA_1c_) lab draw and MPR assessment at enrollment, 3 months, and 6 months. In addition to structured lab draws and MPR assessments, participants had the MAS and “Self-Efficacy for Appropriate Medication Use Scale” (SEAMS) questionnaires completed at baseline and monthly for the 6-month study duration. For additional information on trial design, medication adherence scoring measures, participant recruitment, and sample size determinations, see Multimedia Appendix 1.

### Subject Recruitment

Participants were recruited from the Veterans Affairs Sierra Nevada Health Care System (VASNHCS) between March and November 2021. The VASNHCS is a Veterans Affairs (VA) medical center that provides care to more than 30,000 US military veterans across a large geographical area comprised primarily of rural and highly rural communities. The Veterans Health Administration (VHA) provides comprehensive health and pharmacy benefits to all enrolled veterans. Potential participants were identified and recruited using the VHA corporate data warehouse. Participants older than 18 years with uncontrolled T2DM on active treatment were identified and contacted for participation. Potential participants were recruited in descending priority according to the number of previous HbA_1c_ greater than 8.5% in the last 2 years and were excluded if they were on insulin or were initiated on insulin during the study period. Potential participants were also excluded if they did not own a smartphone or were unable to download the smartphone app and create a user account. There was no formal assessment of technology literacy.

### Standard of Care

The VASNHCS provides comprehensive clinical, specialty, and pharmacy care to all enrolled veterans. Study participants enrolled in the standard of care arm were provided medication reconciliation counseling focused on adherence strategies. They were then instructed to continue to follow up with either their primary care provider or endocrinologist. Participants’ primary care providers and endocrinologists were free to modify their medications, prescribe new medications, and discontinue medications in accordance with the VA national formulary and current clinical practice guidelines.

### Intervention

In addition to medication reconciliation and counseling on medication adherence, participants were provided with a novel smartphone app, DayaMed Arthur (DayaMedicals Incorporated), configured with their current medications. The app provided accurate prompts and reminders to participants to take all medications as directed and paired with caregivers to notify them of medication administration status. The app medication reminders were set up so that they required a participant’s response or action to be satisfied. See Multimedia Appendix 2 for additional information on app reminders and the digital incentivization program.

### Clinical Outcomes

Our primary outcomes of interest were the change in HbA_1c_ compared between the smartphone app intervention arm and the standard of care. Secondary outcomes focused on the evaluation of the difference in proportion of participants able to achieve a clinically meaningful reduction in A_1c_, defined as 0.5%, and the differences in the proportion of participants that required escalation of diabetes medication therapy. Therapy escalation was defined as the addition of a new therapy or an increase in the dose of an existing therapy to the participant’s regimen for the treatment of diabetes. If a participant was converted from a dipeptidyl peptidase-4 inhibitor (DPP4-I) to a glucagon-like peptide-1 (GLP-1) agonist, this was also considered a therapy escalation. De-escalation was considered when a medication dose for the treatment of diabetes was decreased or discontinued. Exploratory outcomes included differences in visits for the management of T2DM as well as the analysis of the variation and changes in MPR, MAS, and SEAMS during each study arm.

### Statistical Methods

We used a modified intent-to-treat approach to complete our analyses. Participants who did not undergo both study arms were excluded from the analyses. Participants were included in the analysis regardless of engagement with the study intervention and care team, provided all lab draws and surveys were completed. The primary end point of the difference in mean reduction HbA_1c_ between the intervention and control groups was evaluated using a paired *t* test. Secondary outcomes of the proportion of participants achieving a reduction of HbA_1c_ of greater than or equal to 0.5% and the proportion of participants requiring therapy escalation were evaluated using McNemar’s test. Other exploratory outcomes included descriptive analysis evaluating the baseline and change in MAS and the change in SEAMS from baseline to completion of each study arm (intervention and standard of care).

### Ethics Approval

This study was reviewed and approved by the University of Nevada, Reno Institutional Review Board (1655381-2). All study participants provided written informed consent before the initiation of any study activities.

## Results

### Study Population

**Figure 1:**
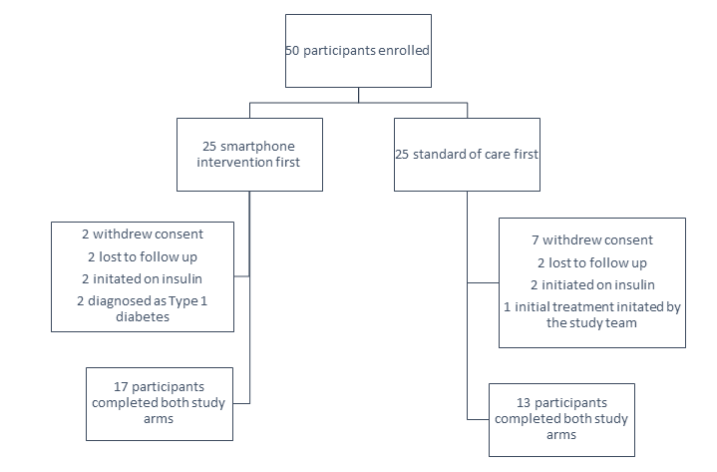
Description of study enrollment and retention.

A total of 50 participants were enrolled over an 8-month period. Out of which 9 participants withdrew consent, 4 participants were lost to follow-up, 4 participants were initiated on insulin, 1 participant was initially diagnosed and initiated on treatment by the study team, and 2 participants were later identified as latent-onset type 1 diabetics. Resulting in 30 evaluable participants (Figure 1) who completed both study arms. Dropouts were twice as high in participants who were enrolled in the standard of care arm first compared to the intervention arm (9/25, 36% vs 4/25, 16%). Only 2 participants dropped out while actively on the app; both traveled frequently and struggled with alerts not adapting to changing time zones. All other participants withdrew during the standard of care arm, primarily due to unwillingness to continue monthly surveys and every 3-month lab draw.

Participants had a median HbA_1c_ of 9.1% (IQR 8.6%-9.6%) and had been living with T2DM for 6 (IQR 3-10) years with a median BMI of 30.8 (IQR 28.2-36.3). Participants had significant comorbidities, most commonly hypertension and hyperlipidemia. The median age of study participants was 66 (IQR 56.25-73.25) years and all were male. Participants were on a median of 8.5 (IQR 6.3-11) medications and 3 (IQR 2-3) medications for the management of diabetes. The most common medication classes for the treatment of diabetes were biguanides (29/30, 96.7%) and sulfonylureas (19/30, 63.3%). New novel medications were also used by participants enrolled in the study, with 6/30, 20% on a GLP-1 agonist and 11/30, 36.7% on a sodium-glucose cotransporter-2 (SGLT2) inhibitor. Of note, all but 1 participant were prescribed statin therapy. Overall, the evaluable participants were similar to the population which dropped out or was excluded from analysis in regard to age, HbA_1c_ at baseline, BMI, MPR, time since first T2DM diagnosis, and comorbidities. The population that dropped out differed on the median number of total medications (5 vs 8.5; *P*=.01). The lower medication burden may have played a factor in participants’ decisions to withdraw from the study. See Multimedia Appendix 3 for the complete study demographic table.

### Review of Clinical Outcome Results

The average HbA_1c_ reduction was 0.69% while participants were in the smartphone app intervention arm versus 0.35% in the standard of care arm (*P*=.30) (Table 1). The SD in both groups was large (0.92% vs 1.30%), pointing toward significant variation in total HbA_1c_ change by participant, which was expected. A total of 21/30 (70%) participants achieved a clinically meaningful reduction in HbA_1c_ of 0.5% in the app intervention arm versus 12/30 (40%) in the standard of care arm (odds ratio 2.29, 95% CI 0.94-5.6; *P*=.09). A total of 16/17 (94.1%) participants in the intervention first arm achieved an HbA_1c_ reduction greater than or equal to 0.5% while on the smartphone app intervention, compared to only 4/13 (30.8%) in the standard of care first arm. A total of 8/13 (72.7%) participants sustained or improved reductions in HbA_1c_ when assigned to the intervention following the standard of care arm. Conversely, only 9/17 (56.2%) participants sustained or improved on HbA_1c_ reductions achieved during the intervention arm when switched to standard of care. Participants had statistically significantly higher odds of a therapy escalation while in the standard of care arm than while on the intervention (n=18/30, 60% vs n=5/30, 16.7%; odds ratio 4.3, 95% CI 1.2-15.2; *P*=.02). Additionally, 3 participants had therapy de-escalated while on the intervention, compared to 0 participants while on the control arm. Therapy escalation was split evenly between participants assigned to the intervention first and the standard of care first (8/17, 47.1% vs 7/13, 53.8%).

**Table 1:** Comparison of intervention outcomes in case-crossover study of a digital intervention versus standard of care.

			Intervention (n=30)		Standard of care (n=30)		*P* value
**Primary effectiveness outcome**							
	Change in HbA_1c_^a^, mean (95% CI)	0.69 (0.36 to 1.02)		0.35 (−0.12 to 0.82)		.30	
**Secondary effectiveness outcome**							
	≥0.5% HbA_1c_ reduction, n (%)	21 (70)		12 (40)		.09	
	Therapy escalated, n (%)	5 (16.7)		18 (60)		.02	
	Therapy de-escalation, n (%)	3 (10)		0 (0)		—^b^	
	Visits for T2DM^c^, median (IQR)	2 (1 to 3)		2 (1 to 3)		—	
**Medication adherence scores**							
	MPR^d^, median (IQR)	112 (100 to 130)		102 (98.5 to 127)		.37	
	RMA^e^, median (IQR)	93.2 (79.3 to 99.1)		—		—	
	Change in MAS^f^, mean (95% CI)	1 (0.48 to 1.52)		−0.10 (−0.54 to 0.34)		.02	
	Change in SEAMS^g^, mean (95% CI)	1.90 (0.86 to 2.94)		−0.20 (−0.56 to 0.16)		<.001	

^a^HbA_1c_: hemoglobin A_1c_.

^b^Not available.

^c^T2DM: type 2 diabetes mellitus.

^d^MPR: medication possession ratio.

^e^RMA: real-time medication adherence score.

^f^MAS: medication administration score.

^g^SEAMS: self-efficacy of appropriate medication use scale.

The median MPR for the 6 months before enrollment was 109% for the study population. During the study intervention arm, MPR was 112% (IQR 100%-130%) and 102% (IQR 98.5%-127%) during the standard of care arm. Despite the MPR maintaining above 100% on average, the median real-time medication adherence (RMA) was estimated to be 93.2% (IQR 79.3%-99.1%). The average baseline MAS was 6 out of 8, defined as moderate adherence, and the baseline SEAMS was 41.5 out of 45, defined as high confidence. The change in MAS (1.0 vs −0.1; *P*=.02) and SEAMS (1.9 vs −0.2 *P*<.001) from baseline to month 3 was higher in the intervention arm compared to standard of care. Overall, there was significant variation between the different adherence scores, though most trended toward moderate to good adherence. MPR and SEAMs appear to report higher adherence rates, whereas MAS and RMA reported moderate adherence rates in study participants. The smartphone app intervention appeared to improve participant MPR, self-reported adherence rates through the MAS, and confidence around medication adherence, as reported by SEAMS. These changes were incremental, but they may represent more significant improvements in true medication adherence.

## Discussion

### Principal Findings

Under conditions of a pragmatic pilot study design, we were able to demonstrate that a novel smartphone medication adherence app demonstrated positive effects in improving medication adherence, patient confidence in their ability to adhere to their medication plan, and blood glucose control in a single-center study among veterans with chronically uncontrolled T2DM. While not achieving statistical significance for our primary end point, the reduction in HbA_1c_ while on the smartphone app trended toward significance, as did the proportion of participants who achieved a clinically meaningful reduction in HbA_1c_. Additionally, participants in the intervention had 4 times lower odds of having an escalation of therapy compared to the standard of care. Given that the study was a cross-over design, therapy escalation in participants initially enrolled in the control arm could have skewed the HbA_1c_ reduction observed in the following intervention arm. However, the escalation of therapy was split evenly between the app first and standard of care first intervention groups. Additionally, HbA_1c_ reduction was consistently observed while participants were on the intervention in both groups. Finally, HbA_1c_ levels appeared to increase when the smartphone app intervention was removed in the app first group, while HbA_1c_ reductions were increased or sustained in the standard of care first group when the smartphone app intervention was added (Multimedia Appendix 4). Collectively, these data corroborate currently published data, suggest that the intervention has a positive impact on lowering blood glucose in patients with complex uncontrolled T2DM, and provide preliminary evidence to support a larger, more definitive study.

An interesting and unexpected finding in the analysis was the difference in therapy escalation and de-escalation between the smartphone app and standard of care observation periods. The overall proportion of participants with an escalation of their diabetes medications during the 3 months of the standard of care arm was 60% (18/30), compared to 17% (5/30) in the intervention arm periods. We were unable to find comparative data with similarly complex participants over a similar duration of follow-up to compare our findings, though this would appear to be a clinically meaningful observation. In a posthoc analysis, we evaluated a matched retrospective cohort (N=60) in which medication escalation was observed in 35/60 58.3% of participants over a 3-month period (Multimedia Appendix 5). This data suggests that the smartphone app intervention may impact therapy escalation, which is meaningful in the management of chronic progressive disease states such as diabetes. A better understanding of the duration of this effect on therapy escalation needs to be evaluated in future studies of this and other adherence interventions.

In our exploratory objective to evaluate different measures of medication adherence, we found significant variation in reported adherence metrics. MPR consistently overestimated adherence and was not able to provide granularity on adherence patterns. RMA provided a more accurate review of medication adherence and an understanding of adherence patterns. Participants’ self-reported adherence scores increased while on the app, which was reflective of patient self-reporting during study visits that the smartphone app increased their confidence in understanding their medication regimen. This finding supports the current literature, which has demonstrated that automated reminders improve participants self-reported adherence scores. In the intervention first cohort, the improvement in self-reported medication adherence scores during the intervention arm was not sustained during the standard of care arm, indicating that perhaps 3 months of app use are not sufficient to build long-lasting habits around medication adherence. It is possible that medication changes during the control arm may have also contributed to reductions in self-reported medication adherence scores. Future research is needed to evaluate the optimal duration of app engagement (or other medication adherence interventions) required to build lasting habits and the impact of new medication changes on established medication adherence habits.

### Limitations

As this was a pilot study, inherent limitations included the small sample size, high rate of dropout, and quasi-experimental cross-over study design. For this pragmatic point-of-care pilot study, we successfully enrolled 50 participants over an 8-month period. Our dropout and exclusion rates were higher than anticipated, with only 30 participants completing the study and being included in the final analysis. We believe the complexity of the population and long-term history of uncontrolled diabetes contributed to the fact that engagement in the standard of care arm was minimal and resulted in 7 of 9 participants withdrawing from the study and 4 participants being lost to follow-up. It is possible that including a nominal incentive in future studies would increase sustained participation in the standard of care arm. Lack of patient engagement in care and commitment to lifestyle modification is a known challenge in the management of patients with chronic disease states, including T2DM. Of note, only 2 participants withdrew consent, and none were lost to follow-up while on the smartphone app. This difference in dropout and lost to follow-up may be due to participants feeling more engaged with their care and care team while using the app, which provides daily alerts, specific notifications, prompts calls from the study team, and a unique nonmonetary incentivization system. Additional research is needed to better understand this difference, as improved engagement using smartphone apps may provide meaningful improvement in health outcomes across populations. With the small sample size of this study, we planned to conduct a cross-over study to ensure comparison groups were balanced in subject complexity. The limitation of this approach is the lack of a truly independent intervention; however, we felt this trade-off was acceptable in this first pilot study and ensured groups were similar for analysis.

Other limitations in our analysis are that the study was conducted in a single center in northern Nevada and limited to the veteran population, and that the treatment and management of diabetes were managed by the participant’s established primary care provider or endocrinologist. While our clinical care setting may not reflect more urban settings, our rural and highly rural population provides new insights into potential solutions for this population, which is significantly underrepresented in research. Deferring the management of diabetes to the care team allowed for variability in prescribing practice to potentially impact the results. The VA has a structured formulary and management guidelines that aim to ensure consistent quality care across the veteran population. We feel that this structure limits the potential impact of significant interprovider variability.

### Conclusions

A novel smartphone app with patient-response-directed provider intervention holds promise in its ability to improve blood glucose control in complex noninsulin-dependent T2DM and is worthy of additional study. This system of intervention may be a viable solution to reduce the medication burden by being a noninvasive method of reducing the need for diabetes medication escalation. RMA provides more granular and actionable detail on patient medication adherence for the care team compared to MPR. A smartphone app with built-in medication alerts can improve patients self-reported medication adherence scores and confidence in taking their medications. However, a 3-month duration of use is not sufficient to build long-lasting improvements in medication adherence or patient confidence.

## Appendix

Multimedia Appendix 1 Additional detailed information related to study design, adherence scoring metrics, and sample size determinations.

Multimedia Appendix 2 Detailed information on application alerts and digital incentivization strategy.

Multimedia Appendix 3 Characteristics of study subjects enrolled in case-crossover study.

Multimedia Appendix 4 Graph of median hemoglobin A_1c_ between study groups.

Multimedia Appendix 5 Matched cohort.

## Abbreviations

DPP4-I: dipeptidyl peptidase-4 inhibitor
GLP-1: glucagon-like peptide-1
HbA_1c_: hemoglobin A_1c_
ISPOR: International Society of Pharmacoeconomics and Outcomes Research
MAS: medication adherence score
MPR: medication possession ratio
PDC: proportion of days covered
RMA: real-time medication adherence
SEAMS: Self-Efficacy for Appropriate Medication Use Scale
SGLT2: sodium-glucose cotransporter-2
T2DM: type 2 diabetes mellitus
VA: veterans affairs
VASNHCS: Veterans Affairs Sierra Nevada Health Care System
VHA: Veterans Health Administration
