# Supplementary material for: Use of a Smartphone-Based Medication Adherence Platform to Improve Outcomes in Uncontrolled Type 2 Diabetes Among Veterans: Prospective Case-Crossover Study
Source: JMIR Diabetes. 2023 Aug 10;8:e44297. doi: 10.2196/44297 (PMC10450533; doi:10.2196/44297)
Supplement: Multimedia Appendix 1 [file diabetes_v8i1e44297_app1.pdf]

Study Design

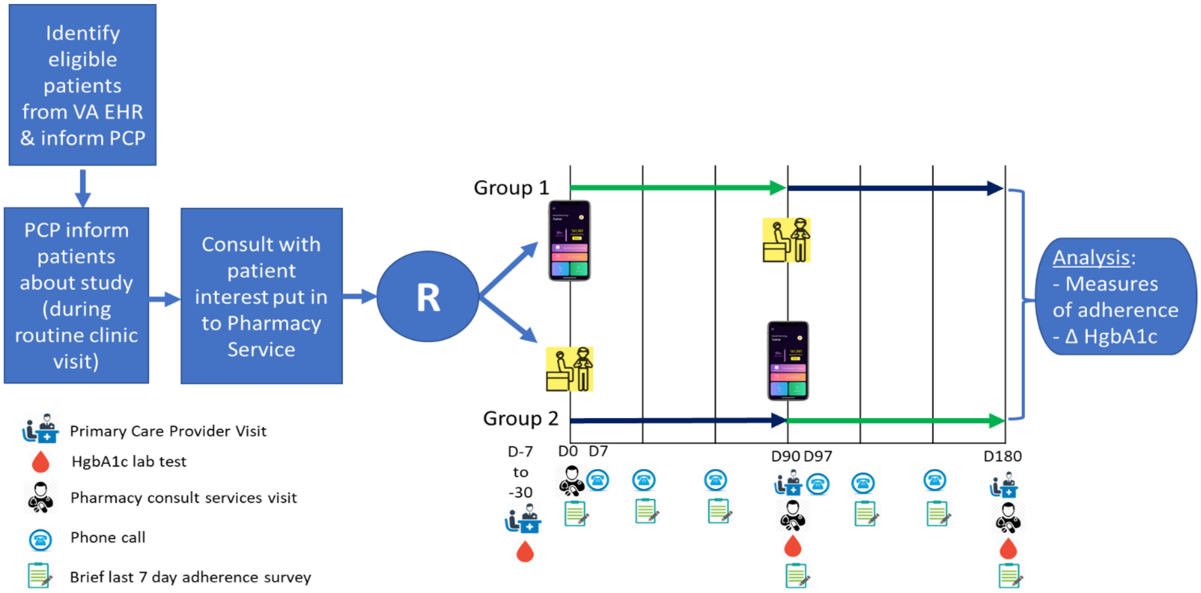

Adherence Scoring Measures

Medication Possession Ratio (MPR) is a standard databased metric for the assessment of medication adherence and is defined as the proportion of a time period where a medication supply is available to be taken by the subject and is determined by the total days supply of a medication dispensed to a subject over a given time period. The Medication Adherence Score (MAS) is an 8-question assessment used to evaluate a subject's adherence to their medication regimen over the past two weeks. Self Efficacy for Appropriate Medication Use (SEAMS) is a 13-question assessment used to evaluate a subject's confidence in their ability to self-manage and adhere to their medication regimen.

Sample Size

Sample size estimates were based on a standard industry benchmark of a minimum clinically meaningful difference of a 0.5% reduction from baseline with an HgbA1c lowering intervention [1,2]. Relevant to a crossover study design with a continuous measurement we considered the following assumptions and sample size requirements. At a significance level (adjusted for sidedness) of 0.025, standard deviation of HgbA1c repeated measures within patients = 0.8 [], a power = 0.8, and a clinically meaningful difference in means before and after therapy of 0.5%, we estimated that a total of 43 patients would be needed to enter this two-treatment crossover study without dropout to achieve the study primary outcome. Assuming a ~20% dropout rate, we estimate that we needed to enroll approximately 50 subjects.

References

1. Ladyzynski, P., Foltynski, P., Bak, M.I. *et al.* Validation of a hemoglobin A_{1c} model in patients with type 1 and type 2 diabetes and its use to go beyond the averaged relationship of hemoglobin A_{1c} and mean glucose level. *J Transl Med* **12**, 328 (2014).
2. Little RR, Rohlfing CL. The long and winding road to optimal HbA1c measurement. *Clin Chim Acta*. 2013;418:63-71. doi:10.1016/j.cca.2012.12.026
3. Selvin E, Marinopoulos S, Berkenblit G, et al. Meta-analysis: glycosylated hemoglobin and cardiovascular disease in diabetes mellitus. *Ann Intern Med*. 2004;141(6):421-431. doi:10.7326/0003-4819-141-6-200409210-00007
