# Supplementary material for: Use of a Smartphone-Based Medication Adherence Platform to Improve Outcomes in Uncontrolled Type 2 Diabetes Among Veterans: Prospective Case-Crossover Study
Source: JMIR Diabetes. 2023 Aug 10;8:e44297. doi: 10.2196/44297 (PMC10450533; doi:10.2196/44297)
Supplement: Multimedia Appendix 2 [file diabetes_v8i1e44297_app2.pdf]

Application reminders

Reminders consisted of push notifications with an alarm at the time a medication was scheduled to be taken and 1 hour after the scheduled administration time if no adherence action had been taken. Adherence actions consisted of subjects entering the DayaMed application and either scanning the barcode of the medication they had taken or selecting the medication from their medication list and bypassing the barcode scan to document adherence. All adherence actions were recorded to create a real time medication adherence score (RMA). In addition to the above daily reminders, email reminders were sent to subjects and the study team when one or more medications was not taken for 3 consecutive days, overall RMA dropped below 80%, or the subject was more than 10 days overdue for a refill. All triggered alerts were acted upon by the study team via a phone call follow up or text message to address any questions, medication adverse effects, or barriers to adherence subjects were experiencing. All medication changes were managed by the subject's primary care team or endocrinologist in accordance with the VA National formulary and current clinical practice guidelines

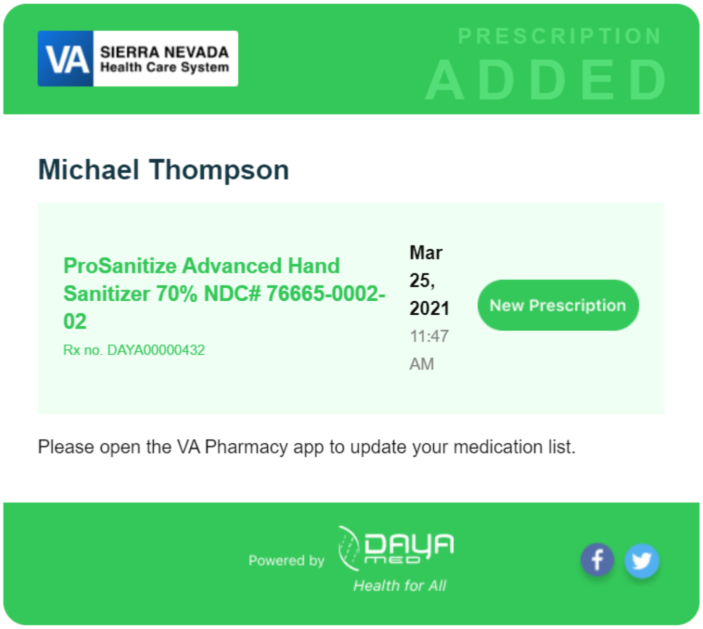

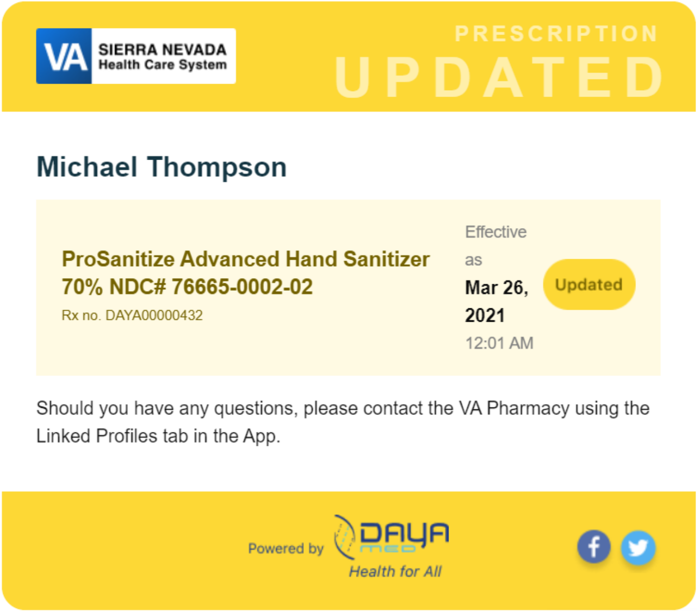

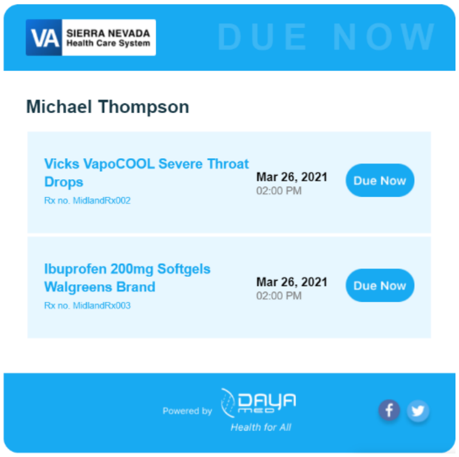

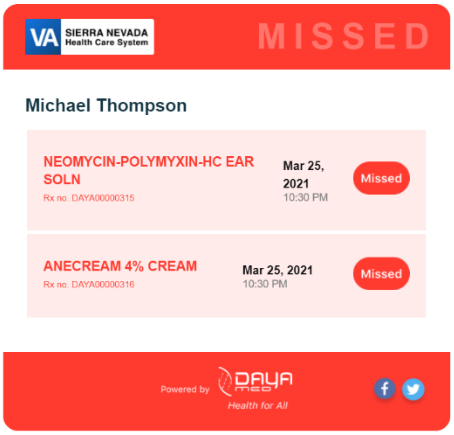

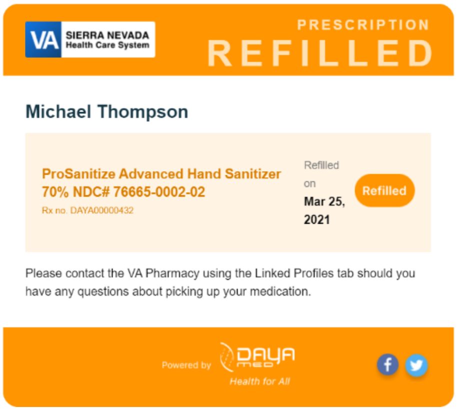

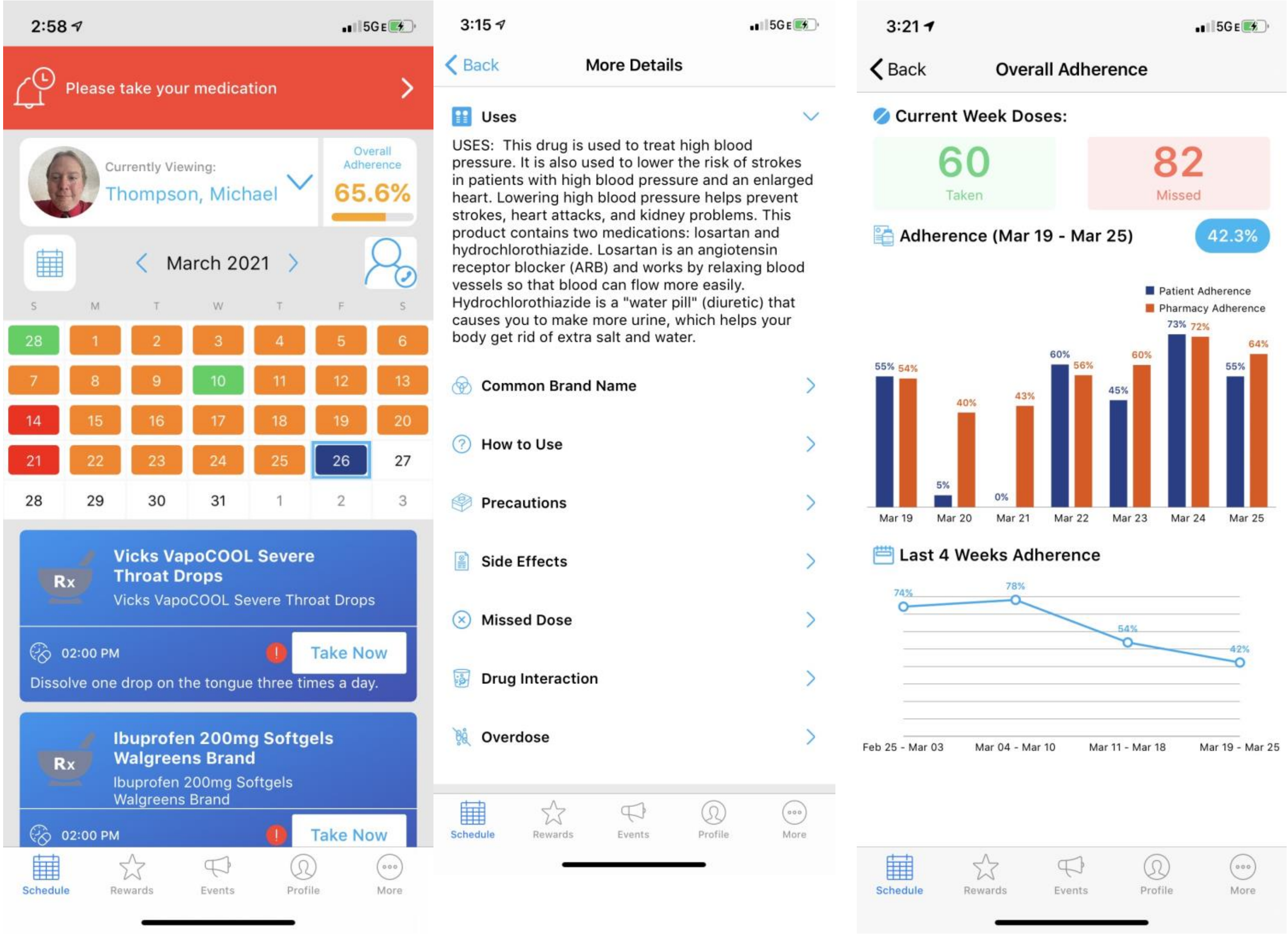

Digital Incentivization Strategy

To create engagement with the application, a challenge-based incentive program was developed. Study subjects were able earn digital “challenge coins” or badges for performing different tasks or achieving specific health targets. These digital incentives were modeled after challenge coins which have a long tradition in the United States military and were earned monthly based on subjects’ cumulative RMA. Subjects could earn bronze, silver, gold, and platinum challenge coins with a new coin being eligible each month. Bronze was earned with an RMA greater than or equal to 80% for one month, silver was earned with an RMA greater than or equal to 90% at two months, gold was earned with an RMA greater than or equal to 95% at 3 months, and platinum was earned by achieving an RMA of 100% for the duration of the study.

Achieved

1

ENROLLMENT

Minimum of 1 prescription needs to be created

Great job, you achieved level 1

2

Achieved

$25

BRONZE

Minimum of 30 days of Participation is required

Great job! You achieved the Bronze adherence level in 115 days

Achieved Date

Mar 03 2021

80.2%

In progress

Schedule Rewards Events Profile More
