# Supplementary material for: Use of a Smartphone-Based Medication Adherence Platform to Improve Outcomes in Uncontrolled Type 2 Diabetes Among Veterans: Prospective Case-Crossover Study
Source: JMIR Diabetes. 2023 Aug 10;8:e44297. doi: 10.2196/44297 (PMC10450533; doi:10.2196/44297)
Supplement: Multimedia Appendix 3 [file diabetes_v8i1e44297_app3.docx]

|  |  | mITT population (N=30) | dropouts (N=13) |
| --- | --- | --- | --- |
| **Demographics** |  |  |  |
|  | Median Age (IQR),years | 66 (56.25-73.25) | 60 (51-73) |
|  | Male, n (%) | 30 (100) | 13 (100) |
|  | Median HbA1c (IQR), % | 9.05 (8.62-9.6) | 9.3 (8.7-10) |
|  | Median Time Since 1^st^ T2DM Diagnosis (IQR), years | 6 (3-10) | 5 (2-9) |
|  | Median BMI (IQR), kg/M² | 30.8 (28.2-36.3) | 32.8 (30.2-37.1) |
|  | Median MPR (IQR), % | 109.0 (100.0-128.8) | 108.0 (97.5-122.0) |
|  | Median medications (IQR) ^b^ | 8.5 (6.25-11.0) | 5.0 (4.0-7.0) |
|  | Median T2DM medications (IQR) | 3.0 (2.0-3.0) | 2.0 (2.0-2.0) |
| **Medications** |  |  |  |
|  | Metformin, n (%) | 29 (96.7) | 12 (92.3) |
|  | Sulfonylurea, n (%) | 19 (63.3) | 7 (53.8) |
|  | alpha glucosidase inhibitor, n (%) | 1 (3.3) | 0 (0.0) |
|  | DPP4, n (%) | 17 (56.7) | 6 (46.2) |
|  | GLP1, n (%) | 6 (20.0) | 2 (15.3) |
|  | SGLT2, n (%) | 11 (36.7) | 0 (0.0) |
|  | Statin, n (%) | 29 (96.7) | 11 (84.6) |
| **Comorbidities** |  |  |  |
|  | CV Event, n (%) | 5 (16.7) | 2 (15.4) |
|  | HTN, n (%) | 20 (66.7) | 8 (61.5) |
|  | HLD, n (%) | 24 (80.0) | 10 (84.6) |
|  | CKD, n (%) | 4 (13.3) | 0 (0.0) |
|  |  |  |  |
|  |  |  |  |

^a^ mITT= modified intent to treat, IQR= interquartile range, HbA1c= hemoglobin A1c, T2DM= Type 2 Diabetes Mellitus, BMI= body mass index, MPR= medication possession ratio, DPP4= dipeptidyl peptidase-4 inhibitor, GLP1= Glucagon-like Peptide-1 Receptor Agonist, SGLT2= Sodium-Glucose Cotransporter-2 inhibitor, CV= cardiovascular, HTN= hypertension, HLD= hyperlipidemia, CKD= chronic kidney disease; ^b^ the difference in the number of medications between groups was statistically significant *(P=*.011), all other differences between groups were not statistically significant
