# Supplementary figures and images for: Use of a Smartphone-Based Medication Adherence Platform to Improve Outcomes in Uncontrolled Type 2 Diabetes Among Veterans: Prospective Case-Crossover Study
Source: JMIR Diabetes. 2023 Aug 10;8:e44297. doi: 10.2196/44297 (PMC10450533; doi:10.2196/44297)

Median HbA1c Results with IQR by Group

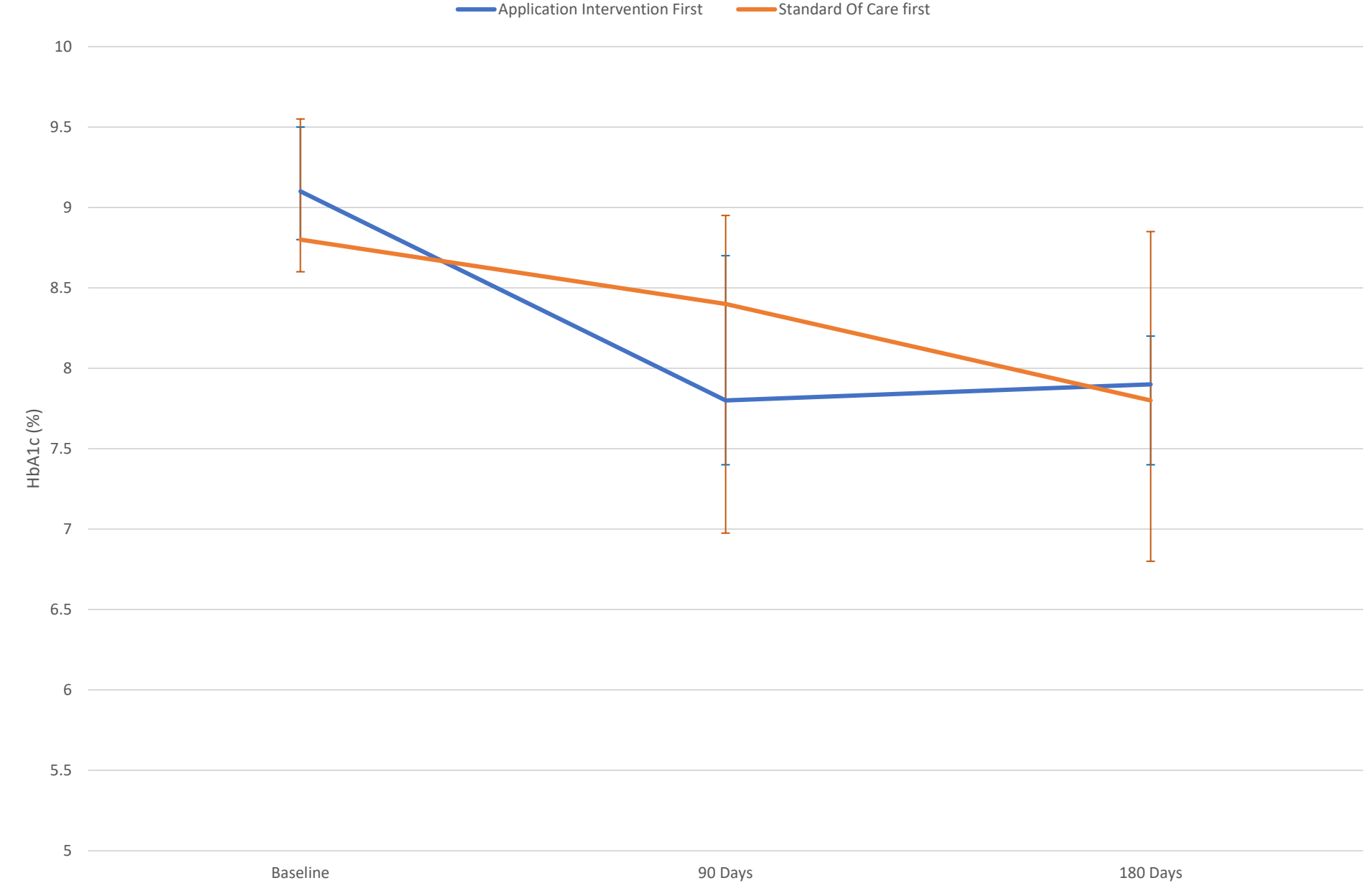

Supplement: Multimedia Appendix 4 [file diabetes_v8i1e44297_app4.pdf]
