# Supplementary material for: Use of a Smartphone-Based Medication Adherence Platform to Improve Outcomes in Uncontrolled Type 2 Diabetes Among Veterans: Prospective Case-Crossover Study
Source: JMIR Diabetes. 2023 Aug 10;8:e44297. doi: 10.2196/44297 (PMC10450533; doi:10.2196/44297)
Supplement: Multimedia Appendix 5 [file diabetes_v8i1e44297_app5.docx]

Appendix A. characteristics of matched cohort for evaluation of therapy escalation rates

|  |  | Matched Retrospective cohort (N=60) |
| --- | --- | --- |
|  |  |  |
| **Demographics** |  |  |
|  | Median Age (IQR), years | 65 (63-70) |
|  | Male, n (%) | 60 (100) |
|  | Median HbA1c (IQR), % | 9.3 (9-9.6) |
|  | Median Time Since 1^st^ T2DM Diagnosis (IQR), years | 6.5 (4-8) |
|  | Median BMI (IQR), kg/M² | 32.1 (29.2-36.2) |
|  | Median MPR (IQR), % | 106.3 (95.8-114.2) |
|  | Median medications (IQR) | 7.0 (6.0-10.0) |
|  | Median T2DM medications (IQR) | 2.0 (2.0-3.0) |
| **Medications** |  |  |
|  | Metformin, n (%) | 55 (91.7) |
|  | Sulfonylurea, n (%) | 33 (55.0) |
|  | alpha glucosidase inhibitor, n (%) | 0 (0.0) |
|  | DPP4, n (%) | 17 (28.3) |
|  | GLP1, n (%) | 8 (13.3) |
|  | SGLT2, n (%) | 20 (33.3) |
|  | Statin, n (%) | 48 (80.0) |
| **Comorbidities** |  |  |
|  | CV Event, n (%) | 13 (21.7) |
|  | HTN, n (%) | 52 (86.7) |
|  | HLD, n (%) | 54 (90.0) |
|  | CKD, n (%) | 2 (3.3) |

^a^ Cohort was created by matching to the mITT population in a 2:1 ratio on age and number of HbgA1c ≥8.5 in the previous 2 years

^b^ mITT= modified intent to treat, IQR= interquartile range, HgbA1c= hemoglobin A1c, T2DM= Type II Diabetes Mellitus, BMI= body mass index, kg/ M²= kilogram/meter², MPR= medication possession ratio, DPP4= dipeptidyl peptidase-4 inhibitor, GLP1= Glucagon-like Peptide-1 Receptor Agonist, SGLT2= Sodium-Glucose Cotransporter-2 inhibitor, CV= cardiovascular, HTN= hypertension, HLD= hyperlipidemia, CKD= chronic kidney disease
